# Quantification of the Mechanical Properties in the Human–Exoskeleton Upper Arm Interface During Overhead Work Postures in Healthy Young Adults

**DOI:** 10.3390/s25154605

**Published:** 2025-07-25

**Authors:** Jonas Schiebl, Nawid Elsner, Paul Birchinger, Jonas Aschenbrenner, Christophe Maufroy, Mark Tröster, Urs Schneider, Thomas Bauernhansl

**Affiliations:** 1 Fraunhofer Institute for Manufacturing Engineering and Automation IPA, 70569 Stuttgart, Germany; 2 Institute of Industrial Manufacturing and Management IFF, University of Stuttgart, 70569 Stuttgart, Germany

**Keywords:** wearable robot, exoskeleton, physical human-robot interaction, physical human–exoskeleton interaction, upper arm, interaction stiffness, interface mechanics, mechanical characterization

## Abstract

Exoskeletons transfer loads to the human body via physical human–exoskeleton interfaces (pHEI). However, the human–exoskeleton interaction remains poorly understood, and the mechanical properties of the pHEI are not well characterized. Therefore, we present a novel methodology to precisely characterize pHEI interaction stiffnesses under various loading conditions. Forces and torques were applied in three orthogonal axes to the upper arm pHEI of 21 subjects using an electromechanical apparatus. Interaction loads and displacements were measured, and stiffness data were derived as well as mathematically described using linear and non-linear regression models, yielding all the diagonal elements of the stiffness tensor. We find that the non-linear nature of pHEI stiffness is best described using exponential functions, though we also provide linear approximations for simplified modeling. We identify statistically significant differences between loading conditions and report median translational stiffnesses between 2.1 N/mm along and 4.5 N/mm perpendicular to the arm axis, as well as rotational stiffnesses of 0.2 N·m/° perpendicular to the arm, while rotations around the longitudinal axis are almost an order of magnitude smaller (0.03 N·m/°). The resulting stiffness models are suitable for use in digital human–exoskeleton models, potentially leading to more accurate estimations of biomechanical efficacy and discomfort of exoskeletons.

## 1. Introduction

Exoskeletons are wearable devices that assist, enhance, or substitute the motor abilities of users [1]. Their applications range from supporting physically impaired individuals or serving as rehabilitation tools to alleviating the physical burden on workers or soldiers during strenuous activities [2,3]. Occupational exoskeletons can reduce muscle and joint strain, aiming to prevent overexertion and long-term musculoskeletal disorders [4,5]. To achieve this, they must apply forces through physical human–exoskeleton interfaces (pHEI) [6]. In addition to supportive forces, undesirable loads may arise at the pHEI, such as parasitic shear forces or torques [7]. These can result from misalignment of human and technical joint axes [7,8], movement restrictions, or design issues [9]. In fact, interaction forces and torques can occur in all directions during movement (see [9,10,11]), with their magnitude largely determined by the exoskeleton’s mechanical architecture [9]. Consequently, efficacy and efficiency decrease when components of the acting force or torque cannot be utilized for joint torque reduction [8] or when energy is lost through tissue deformation (with soft tissues acting as energy sinks) [12,13]. Additionally, these loads can lead to discomfort, pain, or injuries [7,8]. Also, the compliance of soft tissue allows relative movements of body attachments to the human body [14,15,16], which can exacerbate joint axis misalignment and related issues [7,14]. If body attachments slip, shift, or rotate, the entire exoskeleton moves relative to the body, altering lever arms and force directions, thus affecting interaction loads at the pHEI and stresses on joints and muscles. Therefore, the mechanical properties of the pHEI directly influence the efficacy and efficiency of the support, as well as the perception of discomfort [17].

To evaluate the biomechanical efficacy of exoskeletons, experimental methods are employed, such as the investigation of muscle activity or subjective strain [18,19,20]. In addition, musculoskeletal human models (MHM), e.g., the Anybody Modeling System (AMS, AnyBody Technology, Aalborg, Denmark) or OpenSim (National Institute of Health, Bethesda, MD, USA), are utilized, allowing for biomechanical analyses to be conducted earlier during the development process [17]. Furthermore, these models enable the evaluation of parameters that cannot easily be captured experimentally (e.g., joint kinetics) [17]. The models are used to analyze the biomechanical efficacy of exoskeletons [9,21,22,23,24,25,26] or to compare exoskeleton concepts and optimize their design parameters [9,25,27]. As previously described, the mechanical properties of the pHEI directly influence the efficacy and discomfort of the exoskeleton. Consequently, contact modeling in multibody systems significantly impacts the predictive capabilities of simulation models [17].

Often, in such models, loads are applied directly to the rigid body segments (bones) or through rigidly connected structures, offering the least accurate representation of reality [17]. Sometimes, joint degrees of freedom and relative movements are allowed, or in newer approaches, contact elements with adjustable force transmission properties are implemented [9,17,23,26,28]. However, the mechanical properties of the pHEI cannot be defined, which is why other approaches utilize spring-damper models [17]. Model complexity ranges from two-dimensional human models with one-dimensional contact modeling [29,30] to three-dimensional human and contact models [22,31,32]. However, mostly general interaction models have been implemented, without values based on biomechanical measurements (except for Shafiei et al. [32], discussed later), possibly due to a lack of available data. 

In general, the physical interaction between humans and exoskeletons is not well researched, with testing methods varying greatly and lacking harmonization, reproducibility, and realistic testing conditions [6]. So far, supportive normal forces and interaction torques have been measured in pHEI, for example, to recognize user intention and to control active exoskeletons [6,33,34,35,36]. In some cases, shear forces and interaction pressures have been assessed due to their relevance for discomfort and safety [6,10,11,37,38,39,40,41]. However, the mechanical properties of the pHEI have rarely been characterized, even though more accurate MHM could yield more precise estimations of efficacy and discomfort of exoskeleton concepts. Few studies have considered not only forces but also the movements of pHEI [13,32,42,43,44,45]. Mostly, multiple body interfaces were simultaneously and/or multiaxially loaded [42,43,44,45], complicating the characterization of individual pHEI and the application of models in MHM. Fewer studies have considered interaction loads and displacements in isolated loading directions. Langlois et al. determined forces and displacements in an upper-arm pHEI in the arm’s longitudinal direction and were able to derive interaction stiffness and damping parameters [13]. Yet, mechanics of other types of loads (torques) or loading directions (e.g., perpendicular to the arm) were not characterized in any of the studies [13,42,43,44,45]. Shafiei et al. applied forces and torques in different directions to the calf and thigh pHEI and measured loads and displacements with a load cell and motion capture (mocap) to derive a 6 × 6 stiffness tensor [32]. This approach allowed for full-dimensional characterization, although the stiffnesses were approximated linearly [32]. We assume that the anatomy and properties of different soft tissues primarily determine interaction stiffness. Typically, those tissues exhibit non-linear properties, following exponential, polynomial, or power functions [46,47,48]. Such trends were also noted in pHEI (e.g., [42,43]). Since Shafiei et al. applied loads randomly and manually [32], the measurement range for the linear approximation of each stiffness remains unclear. Furthermore, with only three subjects studied, case studies are feasible, but the statistical analysis of mechanical properties is not.

To our knowledge, no pHEI has been mechanically characterized in all direct loading directions with statistical validity. The upper arm interface—vital for shoulder exoskeletons—is particularly under-researched. Also, while there are indications that load–displacement curves progressively increase [42,43], accurate mathematical descriptions are still lacking. Hence, we present a novel methodology that enables the investigation of mechanical pHEI properties under different loading conditions (forces and torques in various axes), allowing controlled and repeatable application of loads to multiple subjects. The loads and displacements of the pHEI are measured simultaneously with high precision to derive mechanical characteristics. Also, relevant factors that could potentially influence the interaction (such as boundary conditions like attachment pressure, muscle activity, interaction speed, and body posture) can be defined within the pHEI, ensuring the acquisition of application-relevant data. We utilize this methodology to examine the upper arm pHEI and derive parameters for use in MHM. Since the mechanical properties of pHEI can be defined in three orthogonal translational and rotational axes in MHM (compare [9,22]), we establish relationships between respective loads and relative displacement in three axes. Hereinafter, we refer to these relationships as “interaction stiffness” for simplicity, even though minor damping and inertia components are included. We characterize non-linear stiffness profiles with mathematical functions that can be easily applied in MHM. We also investigate the magnitudes of interaction stiffness under different loading conditions as well as correlations with anthropometric parameters (e.g., body mass index, arm circumference). Finally, the results are discussed regarding plausibility and applicability to MHM.

## 2. Materials and Methods

### 2.1. Measurement Setup 

**Figure 1:**
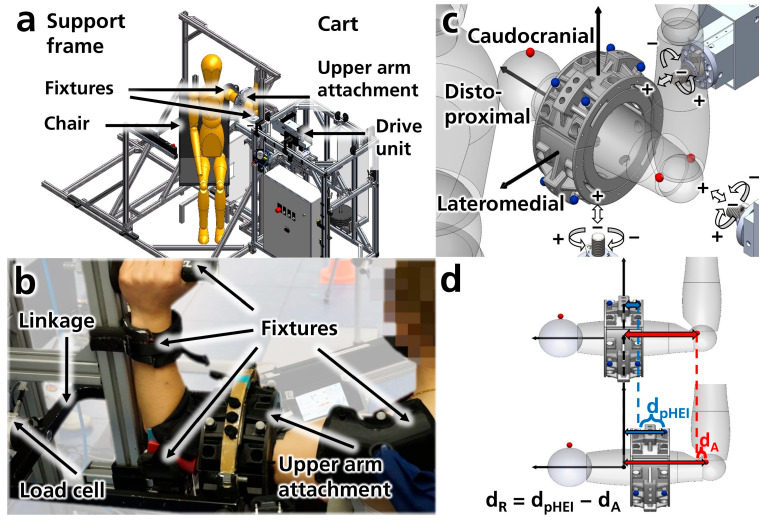
(**a**) Schematic overview of the measurement setup in CAD (computer-aided design, SolidWorks 2021, Dassault Systèmes, Vélizy-Villacoublay, France). (**b**) Subject secured in the setup via fixtures, connected to the support frame. A load cell connects the drive unit and the upper arm attachment (not connected to the support frame) via a linkage. (**c**) Load axes, types, and directions. The setup can exert load types, including forces (white arrows) and torques (white curved arrows) along three orthogonal axes (distoproximal, caudocranial, and lateromedial) in both positive and negative directions at the center of the pHEI. Mocap markers are indicated on the upper arm attachment (blue) and the arm (red). (**d**) The relative motion (d_R_) of the attachment to the arm is calculated as the difference in movement between the pHEI (d_pHEI_) and arm (d_A_) mocap markers.

**Figure A1:**
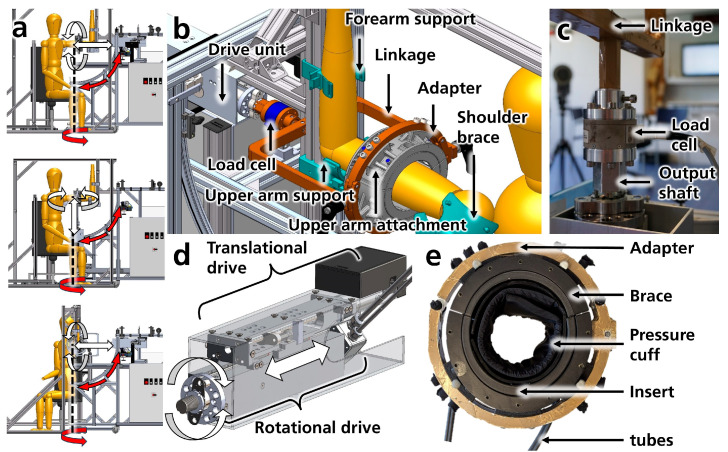
(**a**) A curved rail facilitates the repositioning of the drive unit. At the same time, a vertical axis enables the cart’s rotation (highlighted with red arrows) to achieve various configurations of the measurement setup. These configurations allow torques and forces (white arrows) in the distoproximal (top), caudocranial (middle), or lateromedial (bottom) directions. (**b**) CAD of fixtures (forearm support, upper arm support, shoulder brace), upper arm attachment (pHEI), and drive unit connected to the pHEI adapter via a load cell and linkage. (**c**) Image of the load cell connecting the drive unit’s output shaft and the linkage. (**d**) CAD of drive unit illustrating the translational drive (motor and spindle) moving the rotational drive back and forth, thereby creating translations/forces (white arrows). The rotational drive generates torques around the output shaft (white curved arrows). (**e**) Picture of the pneumatic upper arm attachment, including the adapter, two braces, inserts for circumference adjustment, and pressure cuffs, connected to a pressurized air supply via tubes.

The experimental setup consists of two main structures: a support frame and a cart equipped with a drive unit (Figure 1a). The support frame features various fixtures for positioning and securing the subject’s shoulder and arm, adjustable according to the subject’s anthropometry (Figure 1b). The drive unit can apply both forces and torques (load types) in two directions (positive and negative load directions) to the physical human–exoskeleton interface (pHEI) under investigation (e.g., upper arm attachment, see Figure 1c) via a linkage (Figure 1b). To prevent overloading the subject, several safety mechanisms have been implemented, including a safety controller, mechanical end stops, and emergency switches. Adjustments on the cart allow the application of loads from three orthogonal axes onto the pHEI (Figure 1c, Appendix A.1.2 Figure A1a). The first load axis aligns approximately with the arm’s longitudinal axis (distoproximal axis), while the other two are perpendicular to it: one pointing lateromedially towards the pHEI and the other caudocranially from below. Various pHEI configurations can be tested in this setup. In this instance, a pneumatic upper arm attachment was utilized to control the attachment pressure via padded blood pressure cuffs and a compressed air supply. To measure the forces and torques acting on the pHEI, a load cell (6-axis force-torque sensor K27 × 53, measuring range up to 500 N and 20 N·m, with a maximum measurement error of 1% according to the manufacturer, SENSIX S.A.S., Poitiers, France) is positioned in the linkage between the drive unit and the upper arm attachment (Figure 1b and Figure A1b). Movements of the arm and upper arm attachment are captured using an optical motion capture system with 13 cameras (seven Oqus 700+, four Oqus 400, and one Oqus 310+ for marker capture, and one Oqus 210c for video recording, Qualisys AB, Gothenburg, Sweden). Six mocap markers are attached to the upper arm attachment and the subjects at bony landmarks of the acromioclavicular joint and medial and lateral epicondyles of the humerus (Figure 1b,c). Further details regarding the experimental setup can be found in Appendix A.1.

### 2.2. Measurement Procedure

For the experiments, 21 subjects (10 female, 11 male) aged between 20 and 34 years were recruited from employees at the Fraunhofer IPA. Subjects with sensory disorders or those taking analgesics were excluded from the study. The subjects were informed about the experimental procedures, safety features, and potential risks. Subsequently, their height was recorded, and the length and circumference of the left upper arm (with 90° elbow and shoulder flexion) were measured using a measuring tape. Body mass index (BMI), body weight, body fat percentage, and muscle mass were recorded using a digital body analysis scale (Model HBF-511B-E, OMRON Medizintechnik Handelsgesellschaft mbH, Mannheim, Germany). Mocap markers were attached to the left elbow and shoulder, and arm sleeves (Unisex Armlinge, Brubeck Body Guard, Toronto, ON, Canada) were placed over the upper arm to standardize friction properties and simulate realistic working conditions, as workers typically wear clothing beneath the arm attachment. The subject was positioned in the measurement setup (shoulder: 90° flexion, 45° horizontal extension, 0° rotation; elbow: 90° flexion, 0° pronation/supination), and the setup was adjusted to the first configuration (first selected load axis) to match the subject’s anthropometry. The motion limits of the drives were recorded to prevent collisions between the upper arm attachment and fixtures during the experiments. Additionally, it was ensured (by monitoring a live load cell signal) that the subject did not exert significant pre-tension on the upper arm attachment while in the starting position with a relaxed arm, and this was regularly checked throughout the experiment. Finally, the subject’s arm was secured using the fixtures.

During the experiments, two types of trials were conducted: one experiment to determine discomfort thresholds, which is not presented here, and the subsequently presented trials aimed at assessing interaction stiffness. At the beginning of each trial, we closed the upper arm attachment, told the subject to relax the upper arm, applied a constant attachment pressure of 65 mbar, and initiated an automated measurement in a predefined load axis, type, and direction. The drives applied varying, defined interaction forces between 20 N and 140 N in seven approximately equal increments at a speed of 10 mm/s, or interaction torques between 2 N·m and 10 N·m in five approximately equal increments at a speed of 8°/s. Additionally, a previously determined subject-specific discomfort threshold (range: 0–150 N, 0–10 N·m) was applied, resulting in six or eight different stimuli being presented five times each (in total 30 or 40 stimuli per loading condition). Subjects were first presented with all loads in a defined order, followed by the same loads in four repeated measurements in randomized order. The drives moved the upper arm attachment until the defined load (controlled via predefined current limits) or a predefined motion limit was reached. The movement was held for two seconds, the arm attachment was returned to the starting position, and the next movement was initiated. After completing one set, we repeated measurements for the other load type and direction in the same loading axis. We then rearranged the experimental setup, readjusted it to the subject anthropometry, and repeated the trials in the two other loading axes. The order in which loading axes were selected and the order of loading types and directions within each loading axis were randomized. Short breaks were provided between each change of the loading condition (axis, type or direction), during which the pressure from the upper arm attachment was released, allowing the subject to remove their arm and stand up if needed.

In all trials, movements of the pHEI and arm were recorded using the described camera system (see Section 2.1) and the software Qualisys Track Manager 2023.1 (Qualisys AB, Gothenburg, Sweden). Load cell data were also recorded via Qualisys Track Manager. Additionally, the drives’ positions, velocities, and accelerations were monitored using Elmo Application Studio II (Version Number 2.8.0.22, Elmo Motion Control Ltd., Petah Tikva, Israel). We calibrated the mocap system daily and reset the load cell without the arm present regularly (at least before each new loading condition) to avoid sensor drift and ensure that only interaction forces and torques were measured, excluding the weight of components (upper arm attachment, linkage).

### 2.3. Data Processing and Analysis

We excluded 14 of the 252 measurements (12 loading conditions, 21 subjects) because of missing data (e.g., recording stopped early) or erroneous load cell readings where the measured loads were shifted to unrealistically high values (e.g., baseline a few hundred Newtons above zero, even though no force was applied). The remaining data were imported and processed in an automated workflow using MATLAB (R2023b, The MathWorks, Inc., Natick, MA, USA). Load cell and mocap data were extracted in the respective main loading directions, and relative displacements were calculated as the difference between absolute motions of the arm and arm attachment (Figure 1d). The data were smoothed and subjected to several manual checks, including correcting obviously erroneous data in individual cases. In one instance, force data were offset by ~50 N due to preload during load cell resetting. In five cases, automated starting position detection from mocap data failed, requiring manual definition. Afterwards, the algorithm derived load–displacement curves for each stimulus (one single motion) and carried out an automated offset correction to ensure that force-displacement curves pass the zero point. For each loading condition of each subject, the ten (out of 30 or 40) individual stimuli with the longest load–displacement curves were selected for subsequent analyses of interaction stiffnesses. A detailed outline of the data processing can be found in Appendix A.2.

We fitted various functions to the datasets using the MATLAB function fitnlm() to analyze and mathematically describe the nature of the load–displacement curves. The functions included linear ($f\left(x\right)=ax$), quadratic ($f\left(x\right)=ax^{2}$), and exponential functions ($f\left(x\right)=a{(e}^{bx}-1)$), as well as power laws ($f\left(x\right)=ax^{b}$), all defined to pass through the origin of load and displacement (force/torque: *f*(*x*), displacement: *x*, coefficients: *a* and *b*). We fitted functions to individual stimuli (individual stimulus regression, ISR), to all 10 selected repetitions per loading condition of a subject (subject-specific regression, SSR), and to all selected load–displacement curves of all subjects per loading condition (subject group regression, SGR). We then analyzed the adjusted coefficient of determination R^2^ for each case to facilitate qualitative comparisons of fitting accuracy. The slopes of the fitted first-order polynomials were utilized to compare the magnitudes of interaction stiffnesses across different loading conditions.

## 3. Results

### 3.1. Load–Displacement Profiles

**Figure 2:**
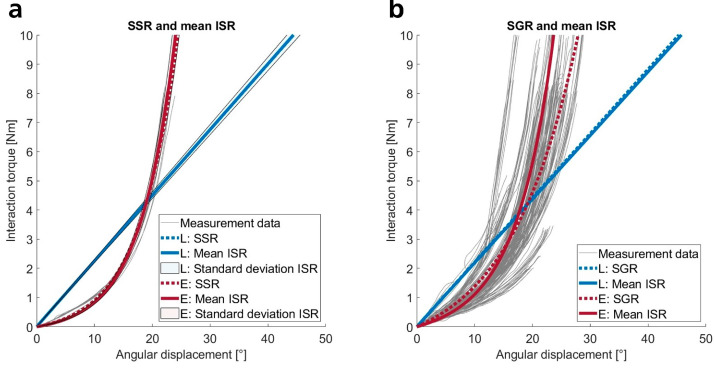
Linear (L) and exponential (E) fittings applied to the measurement data of one subject (**a**) and all subjects (**b**) under a specific loading condition (positive rotation about the lateromedial axis). (**a**) The gray graphs display the ten selected individual stimuli. The means and standard deviations of the corresponding regression functions (ISR) are plotted with continuous lines surrounded by a colored area, while the direct fittings to all ten individual stimuli of one subject (SSR) are plotted with broken lines. (**b**) The figure shows the curves for all the stimuli from all subjects (gray lines), the regression functions fitted directly to this data (SGR, broken blue and red lines), and the means of all ISR fittings (continuous blue and red lines).

Hereinafter, we examine the shape of the load–displacement curves, followed by an analysis of stiffness magnitudes under varying loading conditions. We fitted regression models to the load–displacement data for shape analysis, visually inspected the results, and compared the adjusted coefficients of determination (R^2^) across fittings. Figure 2 displays exemplary load–displacement curves for one subject under a specific loading condition (positive rotation about the lateromedial axis) and for all subjects under the same condition, each fitted with selected regression models. The data reveal that in this loading condition, interaction stiffnesses are more accurately described by exponential functions than by linear models, which is true for both individual subject fits (ISR, SSR) and the fittings to data from all subjects (SGR, see Figure 2).

Table 1 quantifies the loading conditions through the coefficients of determination of the regression models; Figure 2 illustrates one loading condition. The first section of the table lists the mean coefficients of determination for fittings to individual stimuli (ISR). Exponential functions best fit across all directions (mean: R^2^ = 0.99). Power laws and quadratic functions also yield high coefficients (mean: R^2^ = 0.98), while linear models show average R^2^ values of 0.86, indicating a significantly poorer fit than the other models. Notably, straight lines fit the primary data with slightly higher accuracy for rotations around the distoproximal axis and for all translational movements compared to rotations perpendicular to the distoproximal axis. This can be attributed to the steeper ascending load–displacement curves of the rotational movements perpendicular to the distoproximal axis.

We observe similar trends in the analysis of direct fittings per subject and loading condition (SSR). Regression models with exponential functions show the highest coefficients of determination, while straight lines fit the data with less accuracy. Generally, rotational movements are modeled more accurately by exponential functions than translations due to the better fit of exponential functions to the more steeply curved trajectories in rotational movements. Overall, coefficients of determination are slightly lower than those in the ISR. However, the high overall R^2^ values indicate low variability and high repeatability of individual measurements per subject. Visual analysis of the plots further supports this finding, often showing negligible differences between the means of ISR and the corresponding SSR (see, for example, Figure 2a). Standard deviations are also low and often nearly indistinguishable from the mean values. This trend holds across all directions and almost all subjects. In a few exceptions, individual measurement trajectories deviate from the pattern, reducing SSR quality and decreasing R^2^ values.

**Figure A3:**
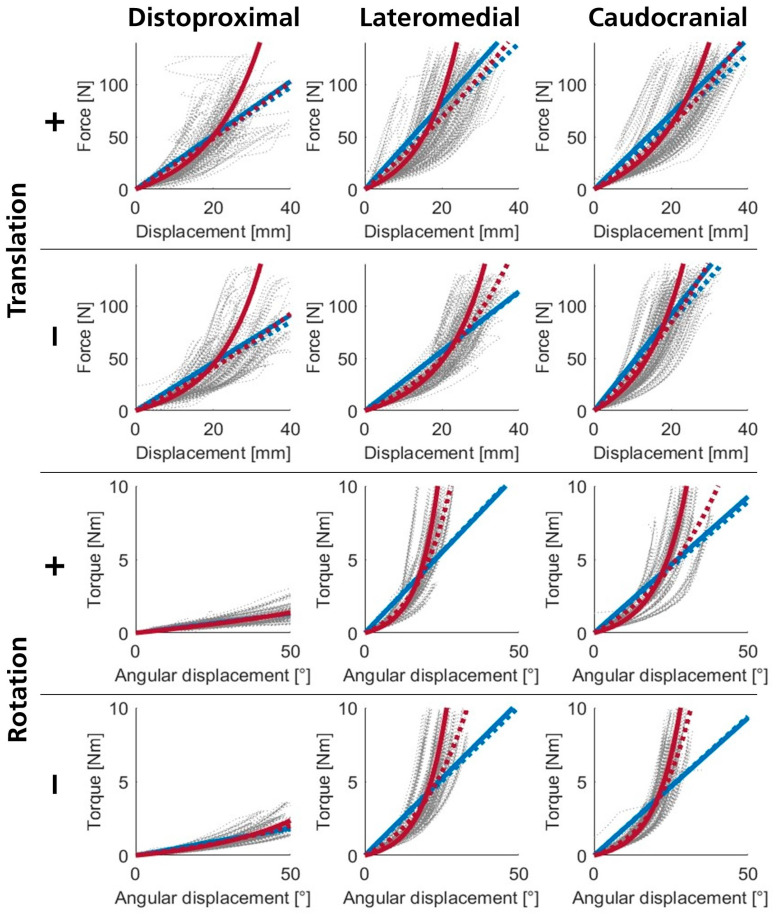
Regression models fitted to measurement data from all subjects (dotted gray lines) for all loading conditions (positive (+) and negative (−) translations and rotations in distoproximal, caudocranial, and lateromedial axes). Fittings to all data are shown as dashed lines (SGR), while continuous lines depict averaged individual stimulus regressions (ISR). Linear regressions appear in blue, and exponential functions are shown in red.

Regression models fitted to subject-specific data (ISR, SSR) describe the qualitative nature of the interaction stiffness of the pHEI. However, fittings for all selected data from all subjects under one loading condition (SGR) may provide universally applicable stiffness models. Figure 2b and Figure A3 (Appendix B.1) present the load–displacement curves of all measurement data and the corresponding SGR. The coefficients of determination in Table 1 are significantly lower compared to ISR and SSR fittings, reflecting notable variability among subjects. On average, coefficients for quadratic, exponential, and power functions are nearly identical and only slightly higher than those for linear regression. R^2^ values for fittings to distoproximal translations are slightly lower due to greater scatter. Figure A3 in Appendix B.1 visually indicates increased variability for these directions as well. Perpendicular rotational loads (lateromedial and caudocranial rotations) exhibit lower variabilities and more pronounced curvature in primary data trajectories. Hence, progressive regression models (Q, E, and P) show slightly higher coefficients of determination for perpendicular rotations compared to other loading conditions (Table 1). The coefficients for the plotted regression models (see Figure A3) are presented in Table A1 (Appendix B.1) for linear and exponential regressions.

We found that SGR fittings often do not accurately represent the qualitative nature of the underlying data due to varying lengths of load–displacement curves across subjects. Smaller displacements yield more data points, dominating the fittings. An alternative could be curves of mean values from individual stimulus regression functions (ISR), presented in Figure A3 and corresponding model coefficients in Table A1 (Appendix B.1). These curves visually match the data better; however, valid R^2^ values are not available. Depending on subsequent modeling questions, one of the two functions may be more beneficial than the other.

### 3.2. Interaction Stiffness Magnitudes

**Figure 3:**
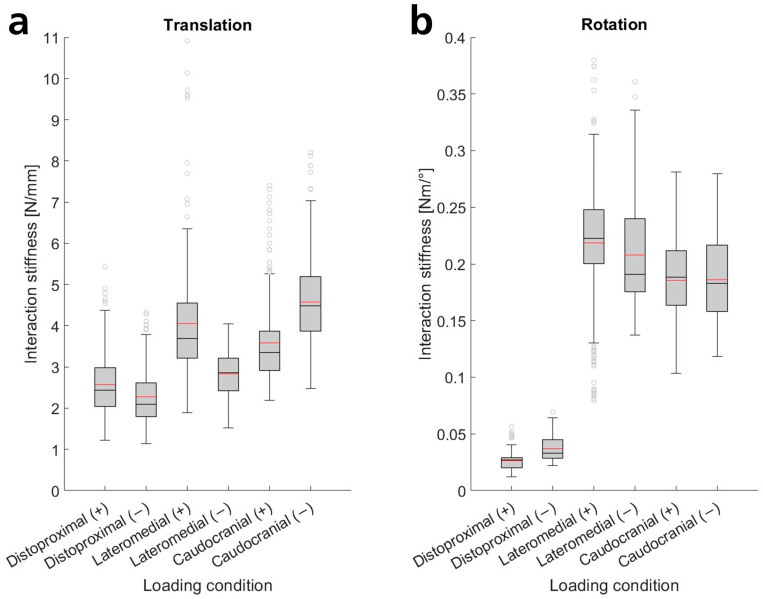
Box plots of the slopes of the ISR linear regression models as a measure of interaction stiffness for the different loading conditions, each with the median (black line), interquartile ranges (gray box), whiskers, and outliers (gray circles). Additionally, the means are indicated (red lines), which can also be found in Table A1 (Appendix B.1). Interaction stiffness values are provided for positive (+) and negative (−) translations (**a**) and rotations (**b**) in all three loading axes (distoproximal, lateromedial, and caudocranial).

To analyze the magnitudes of interaction stiffness and compare different loading conditions, we investigated the slopes of linear regression models, as a single parameter can represent stiffness. The slopes grouped by loading condition mainly did not exhibit a normal distribution (Anderson–Darling test for normality). However, they displayed similarly shaped distributions (tending to be slightly right-skewed). Thus, we performed Mann–Whitney tests to test the inequality of medians across different loading conditions. We found significant differences between almost all loading conditions, except for positive and negative caudocranial rotations relative to each other. Figure 3 presents means and box plots of all ISR linear regression model slopes.

Translational movements exhibited median interaction stiffnesses ranging from 2.1 N/mm to 4.5 N/mm. The lowest stiffnesses occurred in the distoproximal direction, followed by the negative lateromedial direction. The positive lateromedial direction showed significantly higher stiffness. Increased stiffnesses were also observed in the caudocranial direction, with the greatest interaction stiffness in the negative direction (Figure 3, Table A1). The interquartile range was approximately 27–39% of the respective median value, with the greatest deviations in the distoproximal and positive lateromedial directions. The lowest value was 1.1 N/mm, while the highest was 7.0 N/mm without outliers (the highest outlier: 10.9 N/mm).

Interaction stiffnesses for rotational movements can be categorized into two groups. Rotations around axes perpendicular to the arm (lateromedial, caudocranial) exhibited similar stiffnesses (medians between 0.18 N·m/° and 0.22 N·m/°, see Figure 3). Stiffnesses were slightly lower for rotations around the caudocranial axis. Interquartile ranges were between 21 and 34% of the respective median, with lower deviations in positive rotations. The lowest slope determined was 0.10 N·m/°, and the highest was 0.34 N·m/° without outliers (lowest outlier: 0.08 N·m/°, highest outlier: 0.38 N·m/°). The second group, rotational movements around the arm’s longitudinal axis (distoproximal), exhibited stiffnesses reduced by nearly an order of magnitude, with medians of 0.03 N·m/°. Interquartile ranges were 34–49% of the respective median, with the lowest value being 0.01 N·m/° and the highest 0.06 N·m/° without outliers (highest outlier: 0.07 N·m/°). The slopes of the linear regression models and the scatter of the underlying measurement data are also clearly depicted in Figure A3 and quantified in Table A1 (Appendix B.1).

## 4. Discussion

### 4.1. Load–Displacement Profiles

The results show that stiffness profiles in the pHEI progressively increase. Analyses of adjusted coefficients of determination for regression models on individual stimuli (ISR) and per subject (SSR) reveal that exponential functions most accurately fit the data (means of ISR: R^2^ = 0.99, SSR: R^2^ = 0.95), closely followed by quadratic functions and power laws. Visual inspections of graphs confirm this finding, with high similarity in form and trend evident in almost all cases. Only for small movements, especially rotations up to ~10°, do the models slightly underestimate the loads while closely aligning with the rest of the profiles (see Figure 2a). For most simulation-based assessments, these minor differences should not significantly impact. Isolated human soft tissues like skin, fat, and muscle also exhibit progressive stiffness profiles or stress-strain curves, which are flatter at small strains and steeper at higher strains [46,47,48,49]. Consequently, they are often characterized by power laws or quadratic, polynomial, or exponential functions [46,47,48]. This supports the assumption that pHEI stiffnesses are essentially defined by the soft tissue properties. Also, soft tissue stiffness profiles are often simplified using linear (or bi-linear) functions [46,48,49]. In our case, linear approximations did not accurately fit the curves, as expected; however, they still achieved high coefficients of determination, with a mean R^2^ value of 0.86. Fundamentally, linear functions tend to overestimate loads at small displacements and underestimate them at high displacements (Figure 2a). Nevertheless, they serve as a suitable means to intuitively compare interaction stiffness magnitudes, aid in rough calculations, or enable less computationally intensive simulation models. For precise personalized mechanical models of the pHEI, we recommend using exponential functions. However, universally applicable stiffness models would be more practical, as they eliminate the need for new data acquisition for each user.

With an average R^2^ of 0.71–0.76, fitted SGR matched the data less accurately than personalized fittings (ISR, SSR). All non-linear functions exhibited similar coefficients of determination, making them equally suitable for pHEI modeling. However, the models did not ideally follow the load–displacement curves but appeared somewhat too flat. This occurred because varying displacements of individual stimuli resulted in more data points at small displacements being included in the fittings than at high displacements, thus dominating the functions. The averaged ISR across all subjects appeared to follow the natural trend more closely, though it potentially overestimates forces/torques at higher displacements. Depending on the research question or investigation goal, one relationship may be more advantageous than the other, which is why we provide both (see Appendix B.1, Table A1). It should also be noted that in SGR, the differences between exponential (R^2^: 0.75) and linear fittings (R^2^: 0.71) were negligible. Consequently, linear stiffness models can be used within the examined measurement range without significant loss of accuracy. These models are easier to implement, intuitively understandable, and less computationally intensive. They are also suitable for estimating and comparing magnitudes (e.g., for different load directions, arm attachments, or boundary conditions).

### 4.2. Interaction Stiffness Magnitudes

Hereinafter, we interpret stiffness magnitudes before discussing the plausibility of the results. Distoproximal translational stiffnesses were significantly smaller than in perpendicular translations, as shear moduli, which are lower than E-moduli, dominate tissue mechanics (compare muscle tissue [50]). Positive distoproximal stiffness is slightly higher than negative because the upper arm thickens towards the shoulder. Negative lateromedial interaction stiffness is reduced compared to positive, since the relaxed biceps muscle tends to fall slightly medially and can be pushed past the humerus (shear moduli dominate mechanics) without being fully compressed against it, resulting in lower forces at the start of the movement (see Appendix B.1, Figure A3). In the opposite direction, the soft tissue compresses against the bone more quickly, as the humerus tends to lie laterally within the arm volume. Stiffness is lower when pressing against the underside of the arm compared to the top, due to the generally greater presence of adipose tissue in this area [51], which has lower E-moduli [52]. Rotational stiffness around the longitudinal arm axis is lower than for torques perpendicular to the arm, as distoproximal torques are dominated by shear moduli parallel to the muscle fibers. Additionally, lever arms and effective area decrease towards the bone, leading to increased tissue stress and deformation. In torques perpendicular to the arm, however, the tissue is compressed against an approximately constant bone area at the edges of the arm attachment. Positive lateromedial torques exhibit slightly higher stiffness than negative ones, because soft tissue layers are thinner proximally on top and distally on the bottom (see [53]), leading to earlier compression against the bone. Stiffness in caudocranial rotations is lower than in lateromedial rotations, as muscles primarily run above and below the humerus. Thus, in caudocranial rotations, the tissue is partially pushed past the humerus (shear moduli dominate mechanics), whereas in lateromedial rotations, it must be compressed more against the bone. Overall, all measured effects can be plausibly explained with anatomical and tissue knowledge. Additionally, the load cases were simulated in a highly simplified finite element (FE) model of an upper arm with arm attachment to roughly estimate interaction stiffness magnitudes (see Appendix B.2). Both the magnitude and ratios of stiffnesses (e.g., perpendicular vs. distoproximal rotations) were similar to the measured stiffnesses, with a maximum deviation of factor 2, confirming the plausibility of the measured magnitudes.

Comparisons with literature values further support this. Langlois et al. reported average stiffness in the longitudinal upper arm direction ranging from 1.2 to 2.4 N/mm at the same attachment pressure [13]. Despite differing experimental setups and arm attachments, these values align closely with ours (medians: 2.1–2.4). The slightly smaller values in Langlois et al. are likely due to shorter displacement distances (<20 mm vs. >30 mm), as the progressive stiffness curves cause a steeper slope of linear fittings in our study. No additional studies on the upper arm were identified, whereas thigh and calf interfaces have been examined more frequently [32,42,43]. Shafiei et al. determined distoproximal translational stiffnesses of 1.1–3.4 N/mm and perpendicular translational stiffnesses of 2.0–5.3 N/mm [32], closely aligning with our results. Rotational stiffnesses were slightly higher at 0.12–0.15 N·m/° (distoproximal) and 0.25–1.03 N·m/° (perpendicular) [32], likely due to anatomical differences. Since forces and torques were applied randomly and manually, deviations may also arise from varying measurement ranges, as the linear approximation of stiffnesses heavily depends on these ranges (see Section 3.1). Quinlivan et al. investigated stiffness at thigh interfaces, where parallel and perpendicular force components acted simultaneously [43]. At approximately 30 mm displacement, forces between 40 and 80 N were observed [43], yielding linearly approximated stiffnesses of about 1.3 to 2.7 N/mm. Asbeck et al. found similar values in a comparable setup with hip and thigh interfaces [42]. Both studies presented progressive increases in stiffness [42,43]. Although thigh and calf pHEI are not directly comparable to the upper arm, the similar magnitude of results supports the plausibility of the stiffnesses determined here. Furthermore, some approaches measure tissue stiffness rather than total pHEI stiffness directly. Yousaf et al. found tissue stiffness perpendicular to the forearm, ranging from 0.4 to 1.4 N/mm, using indenters [54]. The lower values compared to our study may result from smaller indentations (17 mm vs. >30 mm) and anatomical differences. Mathematical models, such as smooth orthogonal decomposition, have also estimated tissue stiffness from mocap marker trajectories on the calf and thigh, revealing similar translational stiffness magnitudes [55].

The results confirm the plausibility of both the shape and linearly approximated magnitudes of stiffnesses. Next, we discuss our methodology concerning the accuracy of measurement results and the applicability of the determined models.

### 4.3. Limitations and Applicability of Models

The results are subject to the following measurement inaccuracies. The calibrated load cell had a maximum error of 1% of the measurement range (approximately 5 N or 0.2 N·m). Regular load cell resetting ensured that only interaction loads were recorded. The mocap system was calibrated daily, capturing marker positions accurately to the millimeter. However, mocap markers cannot be directly attached to the bone, so skin movement relative to the bone under load may have distorted the measured position of the humerus and the resulting relative displacements by a few millimeters or degrees. Velocities were achieved within fractions of seconds, and the attachment pressure changed only marginally (a few mbar) under load, allowing boundary conditions to be considered constant. Data processing inaccuracies mainly arose from two sources. 

First, minimal pre-tensions applied by the subject in the zero position could not be completely ruled out. However, it was assumed that no load can act at zero relative displacement, so the load–displacement curves were corrected to pass through the origin (minor corrections by a few millimeters or degrees). Second, in distoproximal rotations, absolute rotations were determined instead of relative rotations due to insufficient marker visibility. However, since the arm was very compliant under these loads (large rotations at low torques, see Appendix B.1, Figure A3) and no noticeable rotation of the humerus was observed during experiments, we assume that stiffness was only minimally distorted. Overall, inaccuracies from the measurement setup and data processing are considered minor, having only a marginal impact on the trends and magnitudes of the determined stiffnesses. The analysis of coefficients of determination (SSR) also shows a high repeatability of measurements and reproducibility of the determined stiffnesses. SSR demonstrated a high R^2^ (only slightly reduced compared to ISR), indicating low variation between individual stimuli. Slightly increased variations appeared in the positive translational directions (lower R^2^ of SSR, see Table 1). In these directions, subjects’ arms were less securely fixed (straps instead of rigid fixtures), requiring them to actively counteract motion or correct their posture occasionally. This may have led to brief increases in muscle activity or slight angle changes, affecting interaction stiffness. However, this impacted only a few subjects and often only single stimuli, rendering the impact on stiffness magnitudes and profiles negligible. Nevertheless, future efforts should focus on stabilizing subjects even better to optimize experiment repeatability.

When utilizing our magnitudes and models, note that we have only captured the main elements of a 6 × 6 stiffness tensor; thus, coupling deformations must be neglected when applying the models to rigid multi-body MHM. Furthermore, the “stiffness” results actually describe mechanical impedance, encompassing spring stiffness, damping, and inertia. However, under the investigated constant velocities (no relevant inertia), damping effects were negligibly small (estimated with damping coefficients from [13]). Furthermore, limitations exist in transferring the results to other applications due to the investigated measurement range, subject cohort, arm attachment, and chosen boundary conditions.

Relative displacements exceeding 30° or 30 mm were observed, and the models are expected to be valid within this range, but larger deviations may occur beyond it. The subjects represented body sizes typical of the German population (normally distributed, sizes close to the 5th to 95th percentile according to [56]), with roughly equal numbers of women and men. However, the age was relatively homogeneous, BMI was predominantly within the normal range, and the subjects’ physique did not necessarily match that of workers. In larger or differently composed groups, results may vary more widely or change in level and quality. The boundary conditions were chosen to reflect overhead work. Shoulder and elbow joint angles were aligned with those typical of overhead work [57], and attachment pressures were based on those found in commercial overhead exoskeletons in similar poses (59–72 mbar [58]). Although overhead work heavily strains the shoulder muscles, the upper arm is often minimally affected [21,59], so experiments were conducted with a relaxed upper arm. For other activities, such as heavy lifting or carrying, these boundary conditions may change, and different interaction stiffnesses are anticipated in such scenarios. 

Finally, the results are fundamentally valid only for the specific arm attachment used in the study. To generate accurate models for other arm attachments, experiments must be repeated with those attachments, which is feasible with the current setup. However, since other studies using different upper arm pHEI [13] and even other body interfaces [32,42,43] have produced similar results, it can be concluded that the presented values might be applicable to other upper arm attachments with some loss of model accuracy. In general, the models may be less accurate in predicting outcomes for different users and applications. However, to our knowledge, published values for most loading conditions do not exist, so the functions described here can, for the first time, provide at least a valid magnitude of values for the upper arm interface. Future investigations should aim to quantify how altered boundary conditions and subject cohorts affect the results to derive more universally applicable models, possibly using correction factors or similar approaches. Variation between subjects may be greater than other influences combined. Our investigations have shown that interaction stiffnesses correlate with some easily measurable anthropometric characteristics (BMI, weight, upper arm circumference, gender, see Appendix B.3), which might allow for the future derivation of semi-personalized models that adjust general stiffness models (SGR) using correction functions for individual users.

## 5. Conclusions

In this study, we established a methodology for the comprehensive mechanical characterization of the pHEI under various boundary conditions. We found that the approach allows precise measurement of loads and displacements with high repeatability, yielding plausible interaction stiffnesses. To our knowledge, this is the first time that all diagonal elements of a 6 × 6 stiffness tensor for an upper arm pHEI have been characterized, enabling a full pHEI description in MHM (when neglecting coupling effects). In general, this may be the first instance of a pHEI being fully characterized with enough subjects to obtain statistically significant data. We identified significant stiffness differences between loading conditions. Forces and especially rotations along the arm’s longitudinal axis exhibited notably lower stiffness compared to other conditions. Beyond determining magnitudes, we derived precise mathematical models of pHEI stiffness profiles, best described by exponential functions to capture their non-linear nature. The results enable implementing general and highly accurate personalized interaction models of the pHEI in MHM, applicable to overhead work simulations. If some loss of accuracy is acceptable, our models can also be used for simulations with other arm attachments or application domains.

Future work could optimize the setup to achieve more rigid fixation of subjects. The methodology may be used for further studies to characterize commercial upper arm attachments or explore the impact of various boundary conditions (posture, attachment pressures, relative velocities, muscle activity), populations, or anthropometries on mechanics. Such investigations might foster the development of models with broader general applicability.

## Figures and Tables

**Table 1:** Adjusted coefficients of determination (R^2^) for different fitted regression models. Linear (L) and quadratic (Q) polynomials, as well as exponential functions (E) and power laws (P), were fitted to data from all loading conditions (positive (+) and negative (−) translations and rotations in distoproximal, caudocranial, and lateromedial axes). R^2^ values for the regressions of individual stimuli (ISR) are averaged across all subjects and each ten individual stimuli. Subject-specific regression (SSR) values are averaged across all subjects. The last section shows R^2^ of direct fittings (SGR) to all subject data for each loading condition. The last column presents the mean R^2^ across all loading conditions.

Model	Translation							Rotation						Mean	
	Disto- Proximal		Latero- Medial		Caudo- Cranial			Disto- Proximal		Latero- Medial		Caudo- Cranial			
	+	−	+	−	+	−		+	−	+	−	+	−		
**Individual stimulus regression (ISR)**															
**L**	0.87	0.88	0.84	0.86	0.86	0.88		0.96	0.92	0.81	0.79	0.82	0.80	0.86	
**Q**	0.96	0.99	0.98	0.98	0.99	0.99		0.99	0.99	0.98	0.97	0.97	0.97	0.98	
**E**	0.96	0.99	0.99	0.98	0.99	0.99		0.98	1.00	1.00	0.99	0.99	0.99	0.99	
**P**	0.96	0.99	0.99	0.98	0.99	0.99		0.99	0.99	0.99	0.98	0.98	0.98	0.98	
**Subject-specific regression (SSR)**															
**L**	0.83	0.87	0.77	0.84	0.78	0.87		0.95	0.91	0.80	0.78	0.81	0.79	0.83	
**Q**	0.88	0.94	0.91	0.95	0.91	0.96		0.98	0.97	0.97	0.96	0.96	0.96	0.95	
**E**	0.87	0.94	0.88	0.95	0.89	0.96		0.96	0.97	0.99	0.98	0.98	0.98	0.95	
**P**	0.88	0.94	0.91	0.95	0.91	0.96		0.97	0.97	0.98	0.97	0.97	0.98	0.95	
**Subject group regression (SGR)**															
**L**	0.67	0.67	0.67	0.78	0.72	0.76		0.70	0.75	0.71	0.70	0.71	0.71	0.71	
**Q**	0.67	0.67	0.67	0.84	0.74	0.77		0.70	0.78	0.83	0.78	0.76	0.85	0.76	
**E**	0.67	0.67	0.67	0.83	0.74	0.77		0.70	0.77	0.83	0.77	0.75	0.85	0.75	
**P**	0.68	0.68	0.67	0.84	0.75	0.78		0.70	0.78	0.83	0.78	0.76	0.85	0.76	

**Figure A2:**
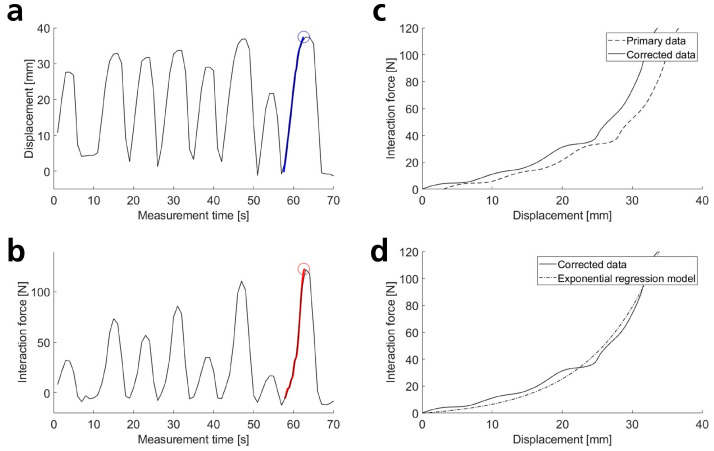
(**a**,**b**) The first 8 of 40 translational movements from one measurement. (**a**) Measured relative displacement of the arm attachment to the arm over time (black line) and a representative slope (blue) with the corresponding maximum (highlighted by a blue circle). (**b**) Measured force on the arm attachment over time and a representative slope (red) with the corresponding maximum (highlighted by a red circle). (**c**) The highlighted force slope from (**b**) is plotted against the displacement slope from (**a**) (measured primary data). A correction determines the distance to the force’s zero-crossing and shifts the curve accordingly, ensuring the corrected trajectory passes through the origin. (**d**) Various regression models are fitted to the curve, illustrated here by an exponential function.

**Figure A4:**
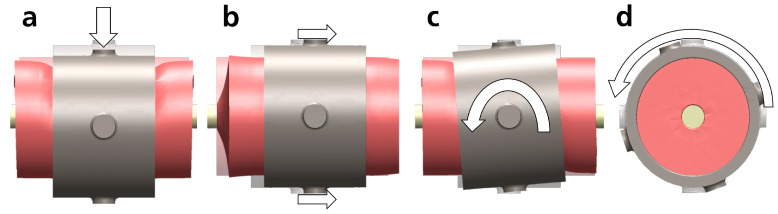
FE-models of simplified arm pHEI under different loading conditions ((**a**) perpendicular force, (**b**) distoproximal force, (**c**) perpendicular torque, (**d**) distoproximal torque). Loads, depicted as white straight arrows representing forces and white curved arrows representing torques, were applied on the surface of the arm attachment (gray), either directly (**d**) or via auxiliary cylinders ((**a**) via one cylinder, (**b**,**c**) via two opposite cylinders for symmetry). The bone (yellow) was fixed to the ground. Deformations of the arm (red) are indicated with respect to the initial position (transparent) and exaggerated for better visibility in (**a**,**b**).

## Data Availability

The raw data supporting the conclusions of this article will be made available by the authors upon reasonable request. To protect privacy, a data transfer agreement is required, and only non-personal data can be shared within the EU.

## Abbreviations

The following abbreviations are used in this manuscript:

mocap	Motion capture
pHEI	Physical human–exoskeleton interface
MHM	Musculoskeletal human models
ISR	Individual stimulus regression
SSR	Subject-specific regression
SGR	Subject group regression
BMI	Body mass index
CAD	Computer-aided design

## Appendix A

### Appendix A.1. Measurement Setup

The measurement setup enables the transmission of forces and torques from a drive unit to a pHEI through a connecting linkage with a load cell (Figure A1c), while the arm is held in place by fixtures. The components and functions of the experimental setup are described in detail below.

#### Appendix A.1.1. Drive Unit

The cart contains a drive unit with a drive axis that can be adjusted between 0° and 90°. This is achieved by pivoting the drive unit along a curved rail around a virtual horizontal axis. Additionally, the entire cart can be rotated 90° around a vertical axis relative to the support frame (Figure A1a). The axes of rotation are aligned so that they intersect at the geometric center of the upper arm attachment in the starting position. In addition to the three described orthogonal loading axes (distoproximal, caudocranial, and lateromedial axes), various combinations of angles theoretically allow for additional loading axes to be set. The cart and drive unit can be accurately positioned and fixed in each configuration.

The drive unit includes a rotational and a translational drive (Figure A1d). The rotational drive consists of an electric motor (EC45, brushless, 250 W, 48 V) with a planetary gearbox (GP52C, 43:1, efficiency: <0.75) and a resolver (Res26, 10 V) from maxon motor GmbH (Munich, Germany), which can generate rotational motion of the output shaft. The drive can produce nominal torques of up to 11 N·m at nominal speeds of approximately 750°/s. The rotational drive is housed in a casing connected to the translational drive via linear ball guides (MINI-RAIL MNNXL 9 G3, Schneeberger GmbH, Höfen an der Enz, Germany). This allows the translational drive to axially displace the output shaft and the rotational drive with minimal friction. The translational drive consists of an electric motor (EC32, brushless, 80 W, 24 V) with a planetary gearbox (GP52C, 14:1, efficiency: <0.75) and a resolver (Res26, 10 V) from maxon motor GmbH (Germany), which drives a ball screw (Carry KGT 10 × 10 FGR RH, diameter: 10 mm, pitch: 10 mm, efficiency: ~0.98, Eichenberger Gewinde AG, Menziken, Switzerland) and can theoretically generate nominal forces of up to 305 N at nominal speeds of about 110 mm/s. In reality, slightly lower nominal forces are achieved since the rotational drive must also be moved, resulting in minor losses due to friction and inertia. The motor control is managed via Elmo Application Studio (EAS) II (Version Number 2.8.0.22, Elmo Motion Control Ltd., Israel) using an external computer with automated motor control scripts that vary from trial to trial.

Since the drives can exert high forces and torques, special precautions were necessary to ensure the safety of the subjects. The movement ranges of both drives are limited by mechanical end stops, and emergency stop buttons are positioned within reach of both the subject and the experiment supervisor. Furthermore, the drives are controlled by safety motion controllers (Platinum Solo Twitter, Elmo Motion Control Ltd., Israel), which achieve a safety performance level of PLe in our setup using resolvers. The controllers can safely limit maximum permissible motor torque (SLT) and deactivate the drives if necessary (STO).

#### Appendix A.1.2. Physical Human–Exoskeleton Interface

The output shaft is connected to an adapter via a connecting linkage (Figure A1b). The adapter can accommodate different body interfaces, such as arm attachments from commercial exoskeletons. In this study, a pneumatic upper arm attachment was used (see Figure A1e), in which the attachment pressure can be adjusted. The 3D-printed upper arm attachment consists of two rigid plastic braces (length: 9 cm). The inner circumference can be adjusted using inserts to fit closely to the circumference of the subject’s arm. Inside, depending on the arm circumference, there are two to three air bladders (dimensions: length 20 cm, width 8 cm) made from blood pressure cuffs (Velcro cuff for children, length 34 cm, width 10 cm, Bosch + Sohn GmbH u. Co. KG, Jungingen, Germany) that cover the entire arm circumference. A textile cover limits the expansion of the cuffs to minimize local bulging. A thin (3 mm) soft foam (Dyafoam laminated with X-Static diamond pattern, Aquilin Röder GmbH, Neusäß, Germany) is placed between the upper arm and the cuff during trials to minimize potential pressure spikes. The cuffs are connected via tubes to a compressed air supply, ensuring the same pressure is applied to all blood pressure cuffs. The compressed air is provided by a compressor (TC-AC 190/6/8 OF, Einhell Germany AG, Landau an der Isar, Germany) and is reduced to the desired output pressure via pressure regulators (Stage 1: pressure regulator RT-M5B, Stage 2: pressure regulator R230-020 with manometer MA6302-C2, AirCom Pneumatic GmbH, Ratingen, Germany).

#### Appendix A.1.3. Support Frame and Fixtures

The support frame consists of a load-bearing aluminum profile where a subject can be positioned and secured. Attached to this frame are several fixtures designed to stabilize the subject’s left arm (Figure A1b). These include a handle, a forearm support, an upper arm support near the elbow, and a shoulder brace. During the trials, the subject sits on a chair, with their upper arm extended forward at 90° shoulder flexion, resting on the upper arm support (see Figure A1b). The forearm support stabilizes the arm against forces in the negative distoproximal direction, while a strap provides stabilization in the opposite direction. Minor support is also provided in the lateromedial direction. The upper arm support primarily provides stability in the negative caudocranial direction, with a corresponding strap offering support in the opposite direction. Lateromedial forces can also be absorbed here. The shoulder is further supported by a shoulder brace, which mainly provides stabilization during negative lateromedial, positive caudocranial, and positive distoproximal translations. The handle helps the subject to actively stabilize against displacements in all directions. All mentioned body interfaces, including the chair, are designed to adjust to different subject anthropometries (circumferences and lengths). The shape and padding of the interfaces were designed to be as comfortable as possible while maximizing the rigidity of the upper arm’s position. This was tested in preliminary trials and iteratively optimized.

### Appendix A.2. Data Processing

During each measurement, eight force or six torque levels were applied and repeated in five sets. In total, 8820 individual stimuli were recorded across 12 movement directions and 21 participants, necessitating automated data processing.

In the first step, data from the load cell (voltage signals from multiple channels), the mocap system (markers’ coordinates), and anthropometric measurements were imported into MATLAB. Force and torque signals were computed using load cell voltages and the calibration matrix provided by the sensor manufacturer. The load in the main loading direction (force in direction or torque about the output shaft axis) was derived and filtered using a Butterworth low-pass filter with zero phase filtering (order: 2; cutoff frequency: 5 Hz for rotations or 0.5 Hz for translations; sampling rate: 100 Hz). A lower cutoff frequency was required for translational movements due to the presence of an overlapping periodic oscillation (~1 Hz), which did not correspond to the participants’ perceived observations and was considered a signal error. However, the filter did not significantly affect the signal progression, as movements typically lasted several seconds (~3–5 s for measurements included in the analysis). Absolute translations along or rotations around the load axis were derived from the motion data of three mocap markers on the arm and four mocap markers at the upper arm attachment, and relative displacements were calculated for subsequent analyses. Displacement data were also filtered (Butterworth low-pass filter with zero phase filtering, order: 2; cutoff frequency: 2.5 Hz; sampling rate: 100 Hz) to remove signal noise without significantly altering the signal progression. During rotations around the longitudinal axis of the arm, not all arm markers were always adequately visible; therefore, the absolute rotation of the upper arm attachment was used as the basis for further evaluations in those cases (instead of the relative displacement). Also, data from the drive, load cell, and mocap were synchronized.

Subsequently, load and displacement curves for all measurements were plotted and reviewed. Fourteen out of 252 measurements were discarded in cases where datasets were incomplete (e.g., due to unnoticed interruptions in recording). In five additional cases, the initial position of the arm connection was not automatically recognized correctly and had to be manually corrected. In the next step, 30 (for rotations) or 40 (for translations) individual stimuli were automatically extracted from each measurement. This involved determining the minima and maxima of the force or torque curves and extracting displacement and load data points between each maximum and the preceding minimum (Figure A2a,b). Since participants still applied slight pre-tensions to the upper arm attachment despite all constructive precautions, the displacement data were automatically corrected (offset correction). The measured displacement at the zero-crossing of the force/torque was determined, and the displacement data were shifted to the origin by this value (Figure A2c). This is reasonable, as it can be assumed that no force/torque can act at zero relative displacement. In cases where no measured zero-crossing was present, the zero-crossing was estimated via a linear fit to the initial segment of the load–displacement curve. The resulting corrected load–displacement curves were subsequently used for fitting and data evaluation (Figure A2d).

## Appendix B

### Appendix B.1. Complementary Data for Overall Stiffness Modeling

**Table A1:** Coefficients (a, b) for fitted linear (L: $f\left(x\right)=ax$) and exponential (E: $f\left(x\right)=a{(e}^{bx}-1)$) regression models. Models were fitted to data from all loading conditions (positive (+) and negative (−) translations and rotations in distoproximal, caudocranial, and lateromedial axes). The table presents the coefficients of fittings to all data (SGR) and to the averaged individual stimulus regressions (mean of ISR curves).

Model	Coeff.	Translation							Rotation					
		Disto- Proximal		Latero- Medial		Caudo- Cranial			Disto- Proximal		Latero- Medial		Caudo- Cranial	
		+	−	+	−	+	−		+	−	+	−	+	−
**Subject group regressions (SGR)**														
I	a	2.4	2.1	3.5	2.8	3.2	4.2		0.03	0.04	0.22	0.20	0.18	0.19
E	a	416	144	297	39	117	148		3.8	1.5	1.0	1.9	3.3	0.9
	b	0.005	0.012	0.010	0.041	0.021	0.022		0.006	0.017	0.085	0.055	0.034	0.081
**Mean of individual stimulus regressions (ISR)**														
I	a	2.6	2.3	4.1	2.8	3.6	4.6		0.03	0.04	0.22	0.21	0.19	0.19
E	a	18	11	12	11	23	21		2.3	0.6	0.3	0.3	0.5	0.4
	b	0.068	0.080	0.106	0.082	0.066	0.088		0.009	0.031	0.156	0.129	0.103	0.120

### Appendix B.2. Comparison of Modeled and Measured Interaction Stiffnesses

To verify the plausibility of the magnitudes of measured interaction stiffnesses, a simplified FE model of a cylindrical upper arm with arm attachment (length: 90 mm) was created in SolidWorks Simulation (SolidWorks 2021, Dassault Systèmes, Vélizy-Villacoublay, France, see Figure A4). Arm and humerus diameters were set at 100 mm and 20 mm, respectively, based on literature [60,61,62]. Bone and arm attachments were assumed to be infinitely stiff. At the same time, soft tissue was modeled as a homogeneous linear elastic material with a shear modulus of 4 kPa and an E-modulus of 22 kPa (muscle tissue according to [50]. Bone and soft tissue were bonded, and soft tissue and arm attachment were either contacted (no penetration contact condition) when simulating perpendicular loads (Figure A4a,c) or bonded, simulating distoproximal loads to enforce tissue deformation without slipping (Figure A4b,d). The position and orientation of the bone were fixed, and forces or torques of 5 N and 0.5 N·m were applied through the arm attachment. Subsequently, displacements were simulated using a solver for large displacements. Under torque loading, rotation angles were calculated based on displacements and trigonometric relationships. Finally, interaction stiffnesses were derived. Simulations were repeated with other loads (1 N, 0.1 N·m) to control for scaling effects.

A translational stiffness (S_mo_) of 6 N/mm was determined perpendicular to the arm (lateromedial and caudocranial) and of 4 N/mm in the distoproximal axis. Rotational stiffness (S_mo_) was 0.11 N·m/° perpendicular to and 0.03 N·m/° around the arm’s longitudinal axis. Measured median translation stiffness (S_me_) was between 24% and 51% (∆) smaller than the corresponding model values S_mo_ (compare Figure 3). Measured values of distoproximal rotation stiffness (S_me_) were 24–40% and perpendicular rotations were 64–100% larger than the corresponding model values ($∆=100\times (S_{mo}-S_{me})÷S_{mo}$). Simulations with lower loads yielded the same results; larger loads could not be solved by the solver due to large displacements.

The models are subject to significant simplifications (linear elastic, anisotropic material model, simplified geometry without different anatomical structures, and small deformations) and should not be used for precise calculations. However, they are suitable for a rough estimation of deformation magnitudes. Modeled and measured stiffnesses are similar and clearly within the same magnitude, emphasizing the plausibility of the results and indicating that interaction stiffness is primarily defined by soft tissue properties. In the future, more detailed FE models (potentially even person-specific) could more precisely approximate the measurement results.

### Appendix B.3. Correlations of Interaction Stiffness and Anthropometry

In addition to the main investigations, correlations (Pearson correlation coefficient) were calculated in Minitab 20.1.2 (Minitab GmbH, München, Germany) between various recorded anthropometric data and interaction stiffnesses (SSR with linear regressions). Correlation coefficients of ±0.3 were interpreted as low, coefficients of ±0.5 as moderate, and coefficients of ±0.7 as strong correlations, following guidelines from medical research [63].

Results are presented in Table A2. We often observed low to moderate correlations between interaction stiffnesses and BMI (7 out of 12), body weight (6 out of 12) or upper arm circumference (6 out of 12). Higher body weight and BMI indirectly promote greater soft tissue thickness, while larger arm circumference directly contributes to this effect, resulting in the tissue being compressed against the bone later. Stiffnesses also correlated with sex, height, and age (3 to 6 out of 12), although these correlations were weaker. These factors are linked to changes in soft tissue composition [51,64,65], which also seems to affect overall stiffness. In contrast, isolated fat and muscle percentages showed low correlations. The results appear plausible and indicate that interaction stiffness is primarily determined by soft tissue thickness and composition.

**Table A2:** Pearson correlation coefficients calculated between interaction stiffnesses of different loading conditions (positive (+) and negative (−) translations and rotations in distoproximal, caudocranial, and lateromedial axes) and anthropometric measures including weight, body mass index (BMI), body fat percentage (Fat), muscle mass (Muscle), sex, upper arm circumference (Circ.), height, and age. Low correlations (>0.3) are highlighted in yellow, while moderate correlations (>0.5) are marked in green.

Var.	Translation							Rotation					
	Disto- Proximal		Latero- Medial		Caudo- Cranial			Disto- Proximal		Latero- Medial		Caudo- Cranial	
	+	−	+	−	+	−		+	−	+	−	+	−
Weight	0.08	−0.37	−0.18	−0.29	0.15	0.43		0.49	0.50	0.14	0.25	0.52	0.57
BMI	0.46	0.04	−0.14	−0.16	0.22	0.44		0.56	0.59	0.16	0.39	0.45	0.40
Fat	0.29	0.30	0.00	−0.20	−0.15	0.27		0.03	−0.03	0.16	0.06	0.12	−0.17
Muscle	−0.14	−0.32	0.02	0.26	0.22	−0.18		0.18	0.21	−0.07	0.11	−0.01	0.28
Sex	−0.09	−0.49	0.07	0.09	0.36	0.24		0.46	0.43	0.10	0.25	0.31	0.53
Circ.	0.07	−0.24	−0.10	−0.20	0.18	0.34		0.59	0.68	0.21	0.40	0.42	0.64
Height	−0.25	−0.45	−0.07	−0.28	0.11	0.28		0.20	0.26	0.16	0.08	0.43	0.62
Age	−0.43	−0.25	0.05	−0.08	0.37	0.43		0.14	0.11	0.00	0.06	0.29	0.33

## References

[1] Bai S., Gurvinder S.V., Sugar T.G. (2018). Wearable Exoskeleton Systems: Design, Control and Applications.

[2] Gull M.A., Bai S., Bak T. (2020). A Review on Design of Upper Limb Exoskeletons. Robotics.

[3] Bogue R. (2015). Robotic exoskeletons: A review of recent progress. Ind. Robot. Int. J..

[4] Bär M., Steinhilber B., Rieger M.A., Luger T. (2021). The influence of using exoskeletons during occupational tasks on acute physical stress and strain compared to no exoskeleton—A systematic review and meta-analysis. Appl. Ergon..

[5] Theurel J., Desbrosses K. (2019). Occupational Exoskeletons: Overview of Their Benefits and Limitations in Preventing Work-Related Musculoskeletal Disorders. IISE Trans. Occup. Ergon. Hum. Factors.

[6] Massardi S., Rodriguez-Cianca D., Pinto-Fernandez D., Moreno J.C., Lancini M., Torricelli D. (2022). Characterization and Evaluation of Human-Exoskeleton Interaction Dynamics: A Review. Sensors.

[7] Näf M.B., Junius K., Rossini M., Rodriguez-Guerrero C., Vanderborght B., Lefeber D. (2018). Misalignment Compensation for Full Human-Exoskeleton Kinematic Compatibility: State of the Art and Evaluation. Appl. Mech. Rev..

[8] Mallat R., Khalil M., Venture G., Bonnet V., Mohammed S. (2019). Human-Exoskeleton Joint Misalignment: A Systematic Review. Proceedings of the 2019 Fifth International Conference on Advances in Biomedical Engineering (ICABME).

[9] Schiebl J., Tröster M., Idoudi W., Gneiting E., Spies L., Maufroy C., Schneider U., Bauernhansl T. (2022). Model-Based Biomechanical Exoskeleton Concept Optimization for a Representative Lifting Task in Logistics. Int. J. Environ. Res. Public Health.

[10] Akiyama Y., Okamoto S., Yamada Y., Ishiguro K. (2016). Measurement of Contact Behavior Including Slippage of Cuff When Using Wearable Physical Assistant Robot. IEEE Trans. Neural Syst. Rehabil. Eng..

[11] Amigo L.E., Fernandez Q., Giralt X., Casals A., Amat J. (2012). Study of patient-orthosis interaction forces in rehabilitation therapies. Proceedings of the 2012 4th IEEE RAS & EMBS International Conference on Biomedical Robotics and Biomechatronics (BioRob 2012).

[12] Young A.J., Ferris D.P. (2017). State of the Art and Future Directions for Lower Limb Robotic Exoskeletons. IEEE Trans. Neural Syst. Rehabil. Eng..

[13] Langlois K., Rodriguez-Cianca D., Serrien B., de Winter J., Verstraten T., Rodriguez-Guerrero C., Vanderborght B., Lefeber D. (2021). Investigating the Effects of Strapping Pressure on Human-Robot Interface Dynamics Using a Soft Robotic Cuff. IEEE Trans. Med. Robot. Bionics.

[14] Schiele A., van der Helm F.C.T. (2006). Kinematic design to improve ergonomics in human machine interaction. IEEE Trans. Neural Syst. Rehabil. Eng..

[15] Jarrasse N., Morel G. (2012). Connecting a Human Limb to an Exoskeleton. IEEE Trans. Robot..

[16] Näf M.B., Koopman A.S., Baltrusch S., Rodriguez-Guerrero C., Vanderborght B., Lefeber D. (2018). Passive Back Support Exoskeleton Improves Range of Motion Using Flexible Beams. Front. Robot. AI.

[17] Scherb D., Wartzack S., Miehling J. (2023). Modelling the interaction between wearable assistive devices and digital human models-A systematic review. Front. Bioeng. Biotechnol..

[18] De Bock S., Ampe T., Rossini M., Tassignon B., Lefeber D., Rodriguez-Guerrero C., Roelands B., Geeroms J., Meeusen R., De Pauw K. (2023). Passive shoulder exoskeleton support partially mitigates fatigue-induced effects in overhead work. Appl. Ergon..

[19] Kopp V., Holl M., Schalk M., Daub U., Bances E., García B., Schalk I., Siegert J., Schneider U. (2022). Exoworkathlon: A prospective study approach for the evaluation of industrial exoskeletons. Wearable Technol..

[20] Mohamed Refai M.I., Moya-Esteban A., van Zijl L., van der Kooij H., Sartori M. (2024). Benchmarking commercially available soft and rigid passive back exoskeletons for an industrial workplace. Wearable Technol..

[21] Fritzsche L., Galibarov P.E., Gärtner C., Bornmann J., Damsgaard M., Wall R., Schirrmeister B., Gonzalez-Vargas J., Pucci D., Maurice P. (2021). Assessing the efficiency of exoskeletons in physical strain reduction by biomechanical simulation with AnyBody Modeling System. Wearable Technol..

[22] Zhou X., Zheng L. (2021). Model-Based Comparison of Passive and Active Assistance Designs in an Occupational Upper Limb Exoskeleton for Overhead Lifting. IISE Trans. Occup. Ergon. Hum. Factors.

[23] Musso M., Oliveira A.S., Bai S. (2022). Modeling of a Non-Rigid Passive Exoskeleton-Mathematical Description and Musculoskeletal Simulations. Robotics.

[24] Schmalz T., Colienne A., Bywater E., Fritzsche L., Gärtner C., Bellmann M., Reimer S., Ernst M. (2022). A Passive Back-Support Exoskeleton for Manual Materials Handling: Reduction of Low Back Loading and Metabolic Effort during Repetitive Lifting. IISE Trans. Occup. Ergon. Hum. Factors.

[25] Tröster M., Wagner D., Müller-Graf F., Maufroy C., Schneider U., Bauernhansl T. (2020). Biomechanical Model-Based Development of an Active Occupational Upper-Limb Exoskeleton to Support Healthcare Workers in the Surgery Waiting Room. Int. J. Environ. Res. Public Health.

[26] Tröster M., Budde S., Maufroy C., Andersen M.S., Rasmussen J., Schneider U., Bauernhansl T. (2022). Biomechanical Analysis of Stoop and Free-Style Squat Lifting and Lowering with a Generic Back-Support Exoskeleton Model. Int. J. Environ. Res. Public Health.

[27] Jensen E.F., Raunsbæk J., Lund J.N., Rahman T., Rasmussen J., Castro M.N. (2018). Development and simulation of a passive upper extremity orthosis for amyoplasia. J. Rehabil. Assist. Technol. Eng..

[28] Seiferheld B.E., Frost J., Krog M., Skals S., Andersen M.S. (2022). Biomechanical investigation of a passive upper-extremity exoskeleton for manual material handling—A computational parameter study and modelling approach. Int. J. Hum. Factors Model. Simul..

[29] Popovic D.B. (1990). Dynamics of the self-fitting modular orthosis. IEEE Trans. Robot. Automat..

[30] Damerau J., Jovic J., Watanabe T., Wolz U. (2015). On the effect of attachment position and compliance of wearable robots on human joint and interface forces. ECCOMAS Thematic Conference on Multibody Dynamics: Proceedings of the ECCOMAS Thematic Conference on Multibody Dynamics 2015.

[31] Zhou X. (2020). Predictive Human-in-the-Loop Simulations for Assistive Exoskeletons. Proceedings of the ASME International Design Engineering Technical Conferences & Computers and Information in Engineering Conference—2020.

[32] Shafiei M., Behzadipour S. (2020). The Effects of the Connection Stiffness of Robotic Exoskeletons on the Gait Quality and Comfort. J. Mech. Robot..

[33] Choi H., Seo K., Hyung S., Shim Y., Lim S.-C. (2018). Compact Hip-Force Sensor for a Gait-Assistance Exoskeleton System. Sensors.

[34] Grosu V., Grosu S., Vanderborght B., Lefeber D., Rodriguez-Guerrero C. (2017). Multi-Axis Force Sensor for Human-Robot Interaction Sensing in a Rehabilitation Robotic Device. Sensors.

[35] Fan Y., Yin Y. (2013). Active and Progressive Exoskeleton Rehabilitation Using Multisource Information Fusion From EMG and Force-Position EPP. IEEE Trans. Biomed. Eng..

[36] Tran H.-T., Cheng H., Lin X., Duong M.-K., Huang R. (2014). The relationship between physical human-exoskeleton interaction and dynamic factors: Using a learning approach for control applications. Sci. China Inf. Sci..

[37] Georgarakis A.-M., Stämpfli R., Wolf P., Riener R., Duarte J.E. (2018). A Method for Quantifying Interaction Forces in Wearable Robots. Proceedings of the 2018 7th IEEE International Conference on Biomedical Robotics and Biomechatronics (Biorob).

[38] Bartenbach V., Wyss D., Seuret D., Riener R., Yu H. (2015). A lower limb exoskeleton research platform to investigate human-robot interaction. Proceedings of the 2015 IEEE International Conference on Rehabilitation Robotics (ICORR 2015).

[39] Wang Y., Qiu J., Cheng H., Zheng X. (2023). Analysis of Human-Exoskeleton System Interaction for Ergonomic Design. Hum Factors.

[40] Ghonasgi K., Yousaf S.N., Esmatloo P., Deshpande A.D. (2021). A Modular Design for Distributed Measurement of Human-Robot Interaction Forces in Wearable Devices. Sensors.

[41] Huysamen K., Bosch T., de Looze M., Stadler K.S., Graf E., O’Sullivan L.W. (2018). Evaluation of a passive exoskeleton for static upper limb activities. Appl. Ergon..

[42] Asbeck A.T., De Rossi S.M.M., Holt K.G., Walsh C.J. (2015). A biologically inspired soft exosuit for walking assistance. Int. J. Robot. Res..

[43] Quinlivan B., Asbeck A., Wagner D., Ranzani T., Russo S., Walsh C. (2016). Force Transfer Characterization of a Soft Exosuit for Gait Assistance. Proceedings of the ASME International Design Engineering Technical Conferences and Computers and Information in Engineering Conference—2015.

[44] Schiele A., van der Helm F.C.T. (2009). Influence of Attachment Pressure and Kinematic Configuration on pHRI with Wearable Robots. Appl. Bionics Biomech..

[45] Schiele A. (2009). Ergonomics of exoskeletons: Objective performance metrics. Proceedings of the World Haptics 2009—Third Joint EuroHaptics conference and Symposium on Haptic Interfaces for Virtual Environment and Teleoperator Systems.

[46] Binder-Markey B.I., Sychowski D., Lieber R.L. (2021). Systematic review of skeletal muscle passive mechanics experimental methodology. J. Biomech..

[47] Fontanella C.G., Toniolo I., Foletto M., Prevedello L., Carniel E.L. (2022). Mechanical Behavior of Subcutaneous and Visceral Abdominal Adipose Tissue in Patients with Obesity. Processes.

[48] Joodaki H., Panzer M.B. (2018). Skin mechanical properties and modeling: A review. Proc. Inst. Mech. Eng. H.

[49] Alkhouli N., Mansfield J., Green E., Bell J., Knight B., Liversedge N., Tham J.C., Welbourn R., Shore A.C., Kos K. (2013). The mechanical properties of human adipose tissues and their relationships to the structure and composition of the extracellular matrix. Am. J. Physiol. Endocrinol. Metab..

[50] Morrow D.A., Haut Donahue T.L., Odegard G.M., Kaufman K.R. (2010). Transversely isotropic tensile material properties of skeletal muscle tissue. J. Mech. Behav. Biomed. Mater..

[51] Weiss L.W., Clark F.C. (1987). Three protocols for measuring subcutaneous fat thickness on the upper extremities. Eur. J. Appl. Physiol. Occup. Physiol..

[52] Singh G., Chanda A. (2021). Mechanical properties of whole-body soft human tissues: A review. Biomed. Mater..

[53] Lanz T.v., Wachsmuth W. (2004). Praktische Anatomie: Ein Lehr- und Hilfsbuch der Anatomischen Grundlagen Ärztlichen Handelns.

[54] Yousaf S.N., Ghonasgi K., Esmatloo P., Deshpande A.D., Kim J. (2022). Human-Robot Interaction: Muscle Activation and Angular Location Affect Soft Tissue Stiffness. Proceedings of the 2022 9th IEEE RAS/EMBS International Conference for Biomedical Robotics and Biomechatronics (BioRob).

[55] Guitteny S., Lafon Y., Bonnet V., Aissaoui R., Dumas R. (2022). Dynamic estimation of soft tissue stiffness for use in modeling socket, orthosis or exoskeleton interfaces with lower limb segments. J. Biomech..

[56] (2010). DIN Deutsches Institut für Normung e. V. Basic Human Body Measurements for Technological Design: Part 2: Statistical Summaries of Body Measurements from Individual ISO Populations.

[57] Maurice P., Čamernik J., Gorjan D., Schirrmeister B., Bornmann J., Tagliapietra L., Latella C., Pucci D., Fritzsche L., Ivaldi S. (2020). Objective and Subjective Effects of a Passive Exoskeleton on Overhead Work. IEEE Trans. Neural Syst. Rehabil. Eng..

[58] Linnenberg C., Weidner R. (2022). Industrial exoskeletons for overhead work: Circumferential pressures on the upper arm caused by the physical human-machine-interface. Appl. Ergon..

[59] De Bock S., Rossini M., Lefeber D., Rodriguez-Guerrero C., Geeroms J., Meeusen R., De Pauw K. (2022). An Occupational Shoulder Exoskeleton Reduces Muscle Activity and Fatigue During Overhead Work. IEEE Trans. Biomed. Eng..

[60] Kumari K.P., Janaki C.S., Lokanadham S. (2022). Midshaft diameter of humerus and its accuracy in sex determination of south Indian population. ijhs.

[61] Singh A., Nagar M., Kumar A. (2014). An Anthropometric Study of the Humerus in Adults. Res. Rev. J. Med. Health Sci..

[62] Gordon C.C., Churchill T., Clauser C.E., Bradtmiller B., McConville J.T., Tebbetts I., Walker R.A. (1989). Anthropometric Survey of U.S. Army Personnel: Summary Statistics, Interim Report for 1988. https://apps.dtic.mil/sti/html/tr/ADA209600/.

[63] Mukaka M.M. (2012). A guide to appropriate use of correlation coefficient in medical research. Malawi Med. J..

[64] Blaak E. (2001). Gender differences in fat metabolism. Curr. Opin. Clin. Nutr. Metab. Care.

[65] Palmer A.K., Jensen M.D. (2022). Metabolic changes in aging humans: Current evidence and therapeutic strategies. J. Clin. Invest..

